# Whole-Genome Shotgun Sequence of *Bacillus mojavensis* Strain RRC101, an Endophytic Bacterium Antagonistic to the Mycotoxigenic Endophytic Fungus *Fusarium verticillioides*

**DOI:** 10.1128/genomeA.01090-14

**Published:** 2014-10-30

**Authors:** S. E. Gold, A. A. Blacutt, R. J. Meinersmann, C. W. Bacon

**Affiliations:** a Toxicology and Mycotoxin Research Unit, U.S. Department of Agriculture, ARS, Athens, Georgia, USA; b Department of Plant Pathology, University of Georgia, Athens, Georgia, USA; c Bacterial Epidemiology and Antibiotic Resistance Research Unit, U.S. Department of Agriculture, ARS, Athens, Georgia, USA

## Abstract

Here, we report the whole-genome shotgun sequence of *Bacillus mojavensis* strain RRC101, isolated from a maize kernel. This strain is antagonistic to the mycotoxigenic plant pathogen *Fusarium verticillioides* and grows within maize tissue, suggesting potential as an endophytic biocontrol agent.

## GENOME ANNOUNCEMENT

*Bacillus mojavensis* strain RRC101 (US patent 5,994,117; ATCC 55732) (1) was isolated from a maize kernel from Northern Italy. It was originally identified as *Enterobacter cloacae* and shown to grow endophytically and enhance the growth of corn seedlings (2). It is antagonistic to *Fusarium verticillioides*, a soil- and seed-borne mycotoxigenic fungus capable of asymptomatic endophytic growth in maize plants. *B. mojavensis* is very closely related to *B. subtilis* (3) but is distinguishable, among other means, by its 16S rRNA sequence (4). The RRC101 genome was prepared as an 8-kb paired-end library and sequenced using 454 technology on a 1/4 run through the Georgia Genomics Facility at the University of Georgia. According to RAST automated annotation of the sequence data and visualization in the SEED viewer, the genome consists of 4,167,987 bp in 4 contigs and includes 4,241 coding sequences with 55 possible missing genes. As there are no public *B. mojavensis* sequences included in SEED, the closest related public genome sequence was that of *Bacillus subtilis* subsp. *subtilis* strain 168 (taxonomy identification no. 224308).

Operons for biosynthesis of antifungal compounds surfactin and fengycin, but not iturin, were identified. Reciprocal BLASTs for antimicrobial lipopeptides, using *Bacillus amyloliquefaciens* FZB42 as a reference, led to identification (>92% identity) of operons corresponding to the surfactin and fengycin biosynthetic pathways. These identifications were bolstered by conserved synteny in the genes within and surrounding these operons. BLAST against the RRC101 genome using the bacillomycin (iturin) operon failed to identify significantly similar genes outside of the already identified fengycin or surfactin pathways, suggesting that RRC101 lacks the PKS-NRPS genes for bacillomycin synthetase and thus likely produces only the fengycin and surfactin class lipopeptides. Preliminary biochemical analyses have indicated that, indeed, several molecular variants of surfactin and fengycin are produced by strain RRC101 (A. A. Blacutt, unpublished data). Additionally, operons for biosynthesis of a bacillibactin-like siderophore, the antibiotic bacilysin, and a number of polyketide synthetase genes similar to those involved in bacillaene production in FZB42 were also identified.

RRC101 possesses a number of genes with likely relevance to rhizosphere competence and endophytism. Specifically, quercetin dioxygenase and acetoin reductase are likely involved in plant-microbe signaling (5), with the former degrading plant-produced antimicrobial root exudates (6) and the latter having implications as producing a known plant growth–promoting volatile compound that likely contributes to the observed enhancement in maize (2) and *Arabidopsis* (M. Rath, unpublished data). Further, whereas the endophytic trait of *B. mojavensis* RRC101 and other bacteria is likely multifactorial, the presence in the genome of genes typically involved in plant colonization, like pectin lyase, expansin, etc., provides mechanistic clues to endophytic competence (7).

### Nucleotide sequence accession number.

The genome sequence of *B. mojavensis* RRC101 has been deposited at NCBI under the accession number ASJT01000101.
